# Discovery of a cryptic aminoacidic triad involved in the temperature adaptation of GH1 enzymes

**DOI:** 10.1111/febs.70515

**Published:** 2026-03-29

**Authors:** Stefania Digiovanni, Gabriel Oanca, Marco Orlando, Marina Lotti, Johan Åqvist, Marco Mangiagalli

**Affiliations:** ^1^ Department of Biotechnology and Biosciences University of Milano‐Bicocca Milan Italy; ^2^ Department of Cell and Molecular Biology Uppsala University, Biomedical Center Sweden; ^3^ Present address: Laboratory for Computational Biochemistry and Drug Design National Institute of Chemistry Ljubljana Slovenia; ^4^ Present address: Laboratory for Environmental and Life Sciences University of Nova Gorica Slovenia

**Keywords:** cold adaptation mechanisms, cold‐active enzymes, empirical valence bond, β‐glucosidase

## Abstract

Cold‐active enzymes exhibit high catalytic activity at low temperatures and an anomalous temperature optimum occurring before the onset of protein unfolding. This study investigates this peculiar property by comparing the functional and structural features of a cold‐active glycoside hydrolase family 1 enzyme (M‐GH1) with those of its mesophilic counterpart (Pp‐GH1). Structural analysis and computational simulations reveal that the thermal profile of M‐GH1 is due to a combination of local unfolding and weakened enzyme‐substrate interactions. This behavior is attributed to the absence of an amino acid triad comprising M326‐W412‐F418, which stabilizes the loops surrounding the active site in the mesophilic enzyme. Introducing this triad into M‐GH1 through rational mutagenesis yielded a more thermostable variant, whereas reciprocal mutations in Pp‐GH1 resulted in a slight decrease of both the optimal temperature of catalysis and thermal stability. Phylogenetic analyses coupled with computational simulations suggest that this flexibility modulation mechanism is not universally conserved across the GH1 family but rather represents a targeted strategy for regulating the dynamics of the catalytic region. Overall, these findings identify a key structural determinant of temperature adaptation within the GH1 family.

## Graphical abstract

Cold‐active enzymes retain high catalytic activity at low temperatures but are characterized by thermal instability, a behavior not fully understood. Comparison between a cold‐active GH1 and its mesophilic counterparts highlights the role of a three‐residue motif (W‐M‐F) in conferring structural stability to a mesophilic GH1. Reciprocal swapping of this motif demonstrated its critical role: It increased thermal stability and optimal temperature in the cold‐active enzyme, while destabilizing the mesophilic counterpart.

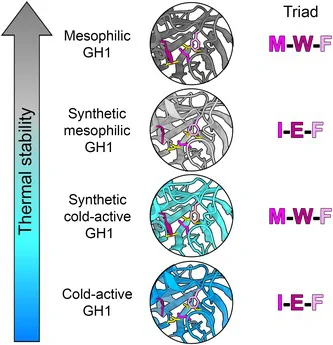

**Abbreviations**
aSAM: atomistic structural autoencoder model
BglU: GH1 from *Micrococcus antarcticus*
GH1: glycoside hydrolases from family 1
ITC: isothermal titration calorimetry
MD/EVB: molecular dynamics‐based empirical valence bond simulations
M‐GH1: GH1 from *Marinomonas* sp. ef1
Pp‐GH1: GH1 from *Paenibacillus polymyxa*
QM/MM: quantum mechanics/molecular mechanics
*T*_*G*_
: temperature gap
*T*_*M*_
: unfolding midpoint temperature
*T*_M(CD)_
: unfolding midpoint temperature measured by CD spectroscopy
*T*_M(FS)_
: unfolding midpoint temperature measured by fluorescence spectroscopy
*T*_*opt*_
: optimal temperature of catalysis

## Introduction

Cold environments cover most of the Earth and are populated by the so‐called psychrophilic organisms. Low temperatures decrease the rate of chemical reactions and would impair metabolic processes [1, 2]. To overcome such issues, these organisms have evolved various strategies, including the production of cold‐active enzymes [3, 4]. A comparison of the functional properties of cold‐active enzymes with those of their mesophilic and thermophilic counterparts has revealed that they typically exhibit high catalytic activity at 5 °C, a low optimal temperature of catalysis (*T*_*opt*_), and thermolability [1, 3, 5]. However, this paradigm is being challenged by the discovery of cold‐active enzymes that combine (high) activity at low temperature with a thermostability comparable to their mesophilic/thermophilic homologs [6, 7, 8, 9]. The only biochemical feature that seems unique to cold‐active enzymes is that the thermal inactivation occurs before the loss of the entire enzyme structure, resulting in a temperature gap (*T*_*G*_) between the unfolding midpoint temperature (*T*_*M*_) and the *T*_*opt*_ [3, 4, 5, 10, 11]. This gap is larger in cold‐active enzymes (19.1 ± 2.7 °C) than in their mesophilic (7.3 ± 1.3 °C) and thermophilic (8.7 ± 1.0 °C) counterparts [11]. Two hypotheses have been postulated to explain this phenomenon: (i) a thermolabile active site that unfolds locally before global unfolding occurs [12, 13, 14], and (ii) a negative heat capacity difference between the transition state and the reactant state of the reaction [15, 16, 17].

From a thermodynamic point of view, the activity of psychrophilic enzymes at low temperatures can be attributed to a redistribution of thermodynamic activation parameters. In particular, cold‐active enzymes exhibit a reduced activation enthalpy (∆H^‡^), which is compensated by a decrease in activation entropy (∆S^‡^). The reduction in activation enthalpy (∆H^‡^) facilitates the formation of the enzyme–substrate transition state at lower temperatures [3, 4, 5, 10, 11, 18]. The structural basis of cold adaptation stems from the higher structural flexibility of cold‐active enzymes compared to their mesophilic and thermophilic counterparts. This enhanced flexibility is achieved through several mechanisms, including the presence of longer and more hydrophilic loops and cavities, the modification of protein topology, the reduction of protein core compactness, and the decrease of intramolecular interactions, as well as a peculiar amino acid composition [3, 4, 5, 10, 11, 18, 19].

Computational simulations provide valuable insights into cold adaptation mechanisms and the temperature dependence of enzyme reactions [18, 20]. These approaches typically combine quantum mechanics/molecular mechanics (QM/MM) calculations with molecular dynamics (MD)‐based empirical valence bond (EVB) simulations [21]. MD/EVB simulations conducted on cold‐active enzymes and their mesophilic counterparts underscore the significance of surface loop flexibility in enzyme cold activity [22, 23, 24, 25, 26]. Notably, these simulations successfully capture the *T*_*opt*_ profile of α‐amylase from the Antarctic bacterium *Pseudoalteromonas haloplanktis*. The simulations reveal that at 25 °C, enzyme–substrate interactions weaken, explaining the temperature‐dependent enzyme inactivation compared to mesophilic enzymes. Additionally, they highlight the role of the β7‐α7 loop region in enzyme temperature adaptation [27, 28].

This study compared the biochemical and structural properties of two glycoside hydrolases from family 1 (GH1): a cold‐active enzyme from the Antarctic bacterium *Marinomonas* sp. ef1 (M‐GH1) [29] and a mesophilic enzyme from *Paenibacillus polymyxa* (Pp‐GH1) [30]. Using a combination of isothermal titration calorimetry (ITC), circular dichroism, and fluorescence spectroscopy, it was shown that M‐GH1 undergoes enzymatic inactivation due to local unfolding events occurring around 30 °C, whereas Pp‐GH1 experiences thermal inactivation due to the loss of its overall structure. MD‐EVB simulations emphasized the significance of interactions between the M326, W412, and F418 side chains in stabilizing the loops surrounding the active site of Pp‐GH1. These interactions are absent in M‐GH1 because of the M326I and W412E substitutions. Rational design mutagenesis has demonstrated that restoring this interaction in the M‐GH1 active site enhances its stability. Conversely, grafting the psychrophilic triad onto Pp‐GH1 yielded a variant with slightly decreased thermal stability.

## Results and discussion

**Fig. 1:**
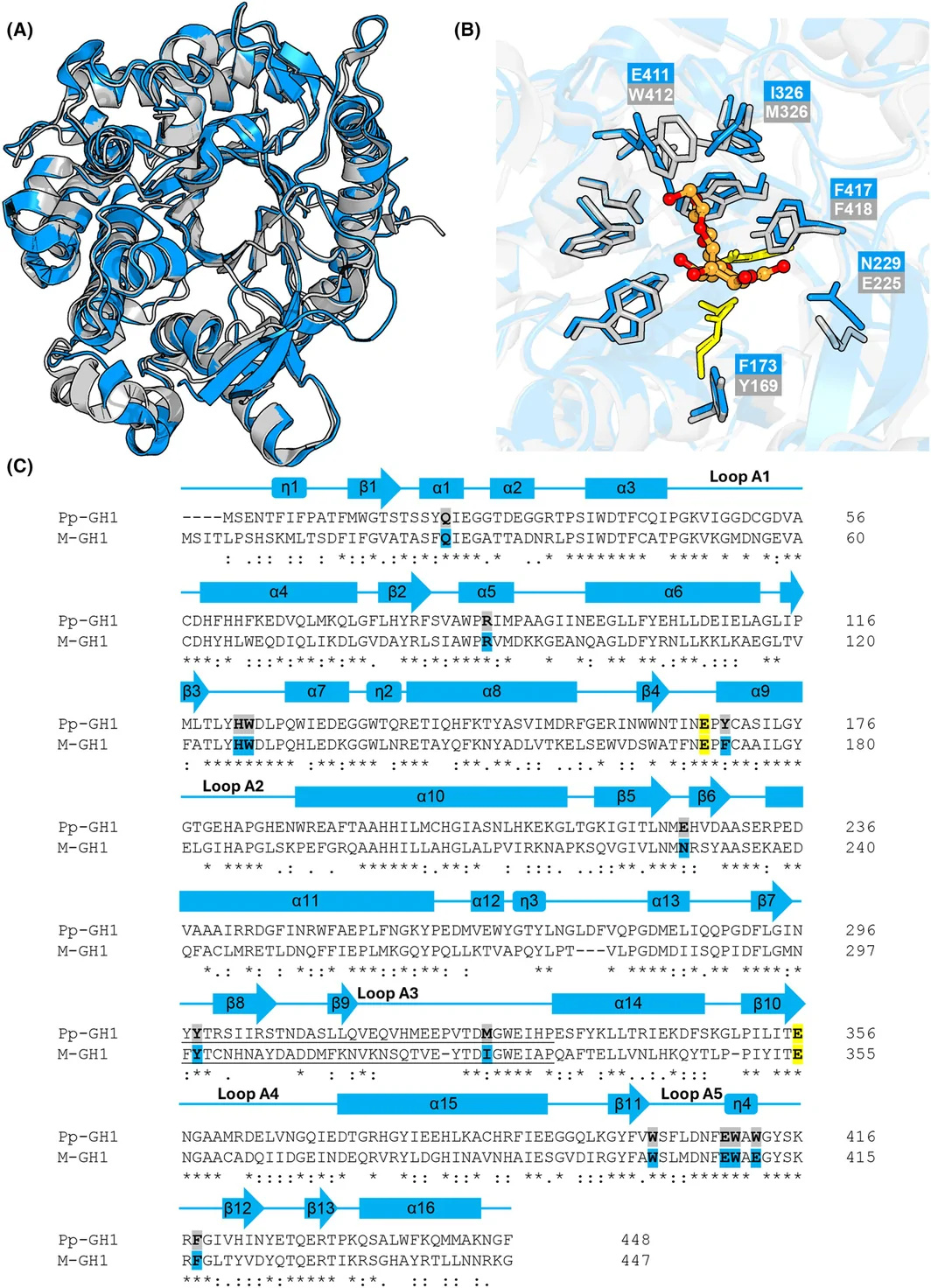
Structural comparison of M‐GH1 and Pp‐GH1. (A) The 3D structure of M‐GH1 (light blue, PDB: 8PUO) superimposed on that of Pp‐GH1 (gray, PDB: 2O9R). (B) Detailed view of the active sites of M‐GH1 (light blue) and Pp‐GH1 (gray). The thiocellobiose molecule cocrystallized with Pp‐GH1 is represented as balls and sticks. The catalytic Glu residues are shown as yellow sticks and the nonconserved residues and conserved F are labeled. Images were prepared using UCSF Chimera X [31]. (C) Structural alignment of M‐GH1 and Pp‐GH1. Active site residues are highlighted with the color code used in panel B. The A3 loops of M‐GH1 (298–331) and of Pp‐GH1 (297–330) are underlined. Sequence alignment was performed using Clustal Omega [32] and manually corrected based on 3D structure comparison. The secondary structure elements were extracted from PDB: 8PUO.

The cold adaptation mechanisms of enzymes are typically elucidated by comparing the biochemical and structural properties of cold‐active enzymes with those of their mesophilic and/or thermophilic counterparts [12, 13, 18]. In this study, we focused on two glycosidases from the GH1 family with available 3D structures: M‐GH1 from the Antarctic bacterium *Marinomonas* sp. ef1 [29] and Pp‐GH1 from mesophilic *Paenibacillus polymyxa* [30]. These enzymes share 45.4% amino acid sequence identity, with their active sites constituted by two glutamate residues (E171 and E355 in M‐GH1, E167 and E356 in Pp‐GH1) surrounded by five loops (A1‐5). Structural alignment revealed good structural conservation between M‐GH1 and Pp‐GH1, particularly in the active site region (Fig. 1). Within this region, only four M‐GH1 residues (F173, N229, I326, and E411) are not conserved, being substituted in Pp‐GH1 by Y169, E225, M326, and W412, respectively. Both enzymes exhibit comparable length of the A3 loop (residues 298–331 in M‐GH1 and 297–332 in Pp‐GH1), despite low residue conservation within this loop (Fig. 1). The A3 loop length varies among GH1 enzymes and appears to contribute to M‐GH1's cold activity [29].

### M‐GH1 is a true cold‐active enzyme whose inactivation depends on the thermolability of the active site

The effects of temperature on kinetics parameters (*K*_M_ and *k*_*cat*_) were investigated using ITC analyses in single‐injection mode and pNPGlc as a substrate. This technique enables fine control of the reaction temperature and continuous monitoring of the reactions, which is not achievable with conventional GH substrates such as *p*‐nitrophenyl glycosides or natural oligosaccharides [33, 34].

**Fig. 2:**
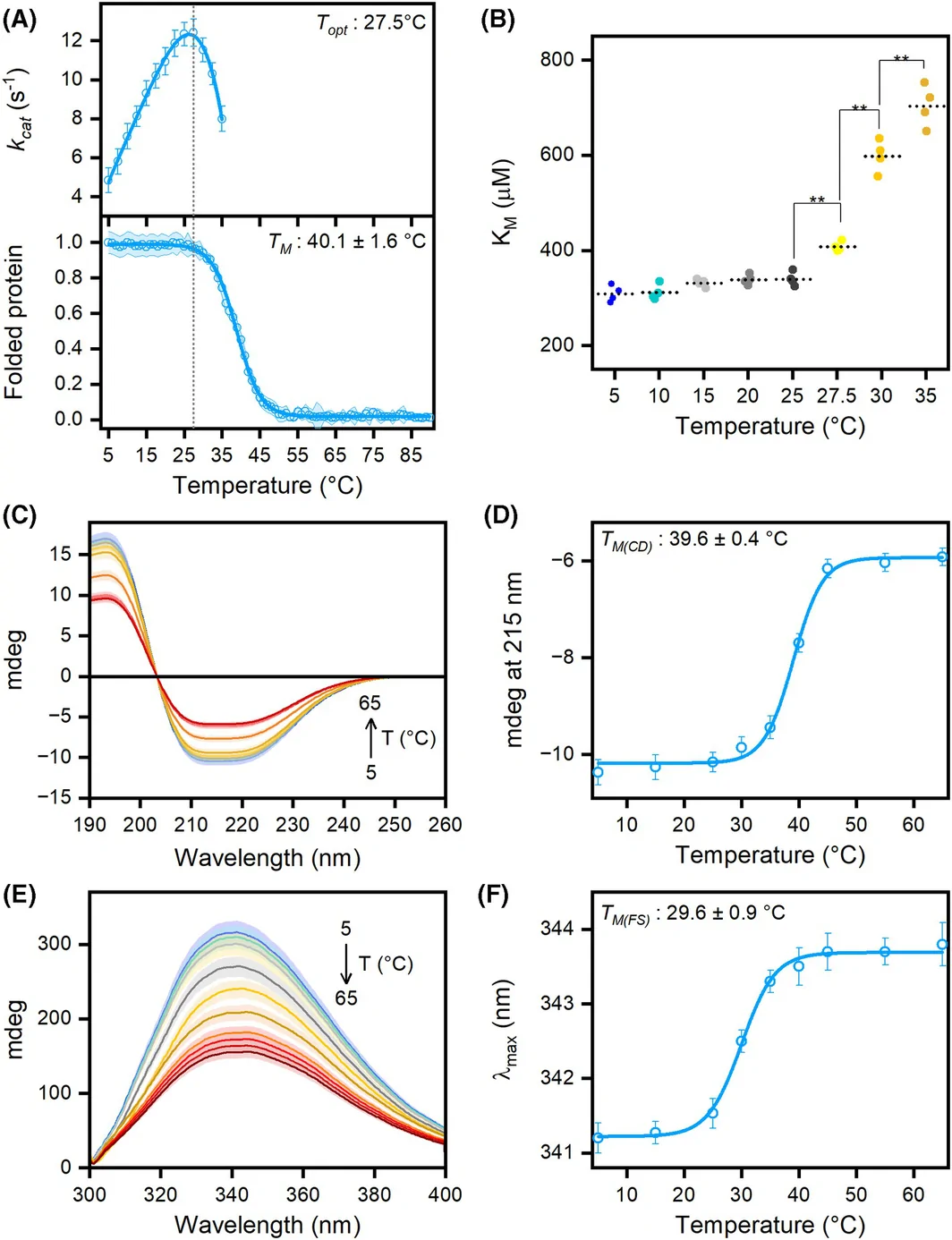
Effects of temperature on the function and structure of M‐GH1. Effects of temperature on *k*_*cat*_ (A, upper panel) and *K*_M_ (B) of M‐GH1 determined by ITC analysis. Reactions were performed in a 25 mm phosphate buffer (pH 7.0) using pNPGlc as a substrate (final concentration, 5.0 mm) at an injection rate of 35 μL·s^−1^. The thermal stability of M‐GH1 (A, bottom panel) was determined by CD spectroscopy. Ellipticity values were recorded at 215 nm for M‐GH1 during heating from 5 to 90 °C. The initial CD signal was considered to be 100% for normalization. Effects of temperature on the structure of M‐GH1 determined by CD spectroscopy (C, D) and intrinsic fluorescence spectroscopy (E, F). CD spectra (C) and fluorescence emission spectra (E) of M‐GH1 measured after ITC analyses were performed by injecting 25 mm phosphate buffer (pH 7) at an injection rate of 35 μL·s^−1^ instead of pNPGlc. The thermal denaturation plot was obtained by plotting the CD signal at 215 nm (D) or the wavelength of the maximum fluorescence emission peaks (F) as a function of temperature. Experiments were performed in quadruplicate, and the error bars or shaded areas indicate the standard deviation (*n* = 4). Statistical analyses were performed using an unpaired two‐tailed Student's *t*‐test, **p < 0.01.

M‐GH1 exhibited highest catalytic activity at 27.5 °C (*k*_*cat*_: 12.8 ± 0.3 s^−1^), while it maintained 37.5% of its activity at 5 °C (Fig. 2A). Above 35 °C, the thermograms became flat and noisy, suggesting enzyme inactivation (data not shown). The thermal denaturation experiments indicate that M‐GH1 loses its secondary structure at temperatures above 35 °C with a *T*_*M*_ of 40.1 ± 1.6 °C (Fig. 2A). For M‐GH1, the *T*_*G*_ is 12.6 °C, close to the average *T*_*G*_ value observed for cold‐active enzymes (19.1 ± 2.7°C) and higher than the average values for mesophilic (7.3 ± 1.3 °C) and thermophilic (8.7 ± 1.0 °C) enzymes [11]. Interestingly, the *T*_*opt*_ determined by ITC analysis is lower than that determined spectrophotometrically [29]. This discrepancy may be due to differences in experimental conditions: ITC measurements involve longer reaction times (40 min) and continuous stirring, whereas spectrophotometric assays are performed statically for shorter durations (5 min).

To test the hypothesis that ITC measurements affect the structure of M‐GH1, CD and fluorescence spectroscopies were conducted on samples obtained following the ITC measurement. In these experiments, PB was injected into the sample cell instead of pNPGlc. The CD spectrum of M‐GH1 measured after the ITC analysis at 5 °C (blue line in Fig. 2C) was superimposable with that obtained from freshly purified enzymes. It presented two minima peaks at 210 and 222 nm, typical of proteins containing *α*/*β* secondary structures [29]. M‐GH1 maintained its secondary structure up to 35 °C, and at temperatures > 40°C, a flattening of the CD signal was observed (Fig. 2C). The *T*_M(CD)_ was determined by fitting the CD signal measured at 215 nm as a function of the temperature, yielding a value of 39.6 ± 0.4 °C (Fig. 2D). The *T*_M(CD)_ value is similar to *T*_*M*_, indicating that ITC incubation does not affect the secondary structure of M‐GH1. The fluorescence spectrum of M‐GH1 at 5 °C exhibited a maximum at 341.2 nm. With increasing temperature, a red shift was observed, accompanied by a decrease in fluorescence intensity (Fig. 2E). The *T*_M(FS)_, determined by fitting the *λ*_max_ as a function of temperature, is 29.6 °C (Fig. 2F), which is 10 °C lower than the *T*_M(CD)_. Given the proximity of the W residues to the active site, we hypothesize that they become more exposed to the increasing temperature, causing local unfolding. Taken together, our data suggest that the early catalytic inactivation observed during ITC analyses results from local unfolding events rather than a loss of overall enzyme structure. These findings support the general mechanisms proposed by Feller and Gerday in the early 2000s to explain the wider *T*_*G*_ observed in psychrophilic enzymes compared to their mesophilic and thermophilic counterparts [3, 12, 13, 14].

**Fig. 3:**
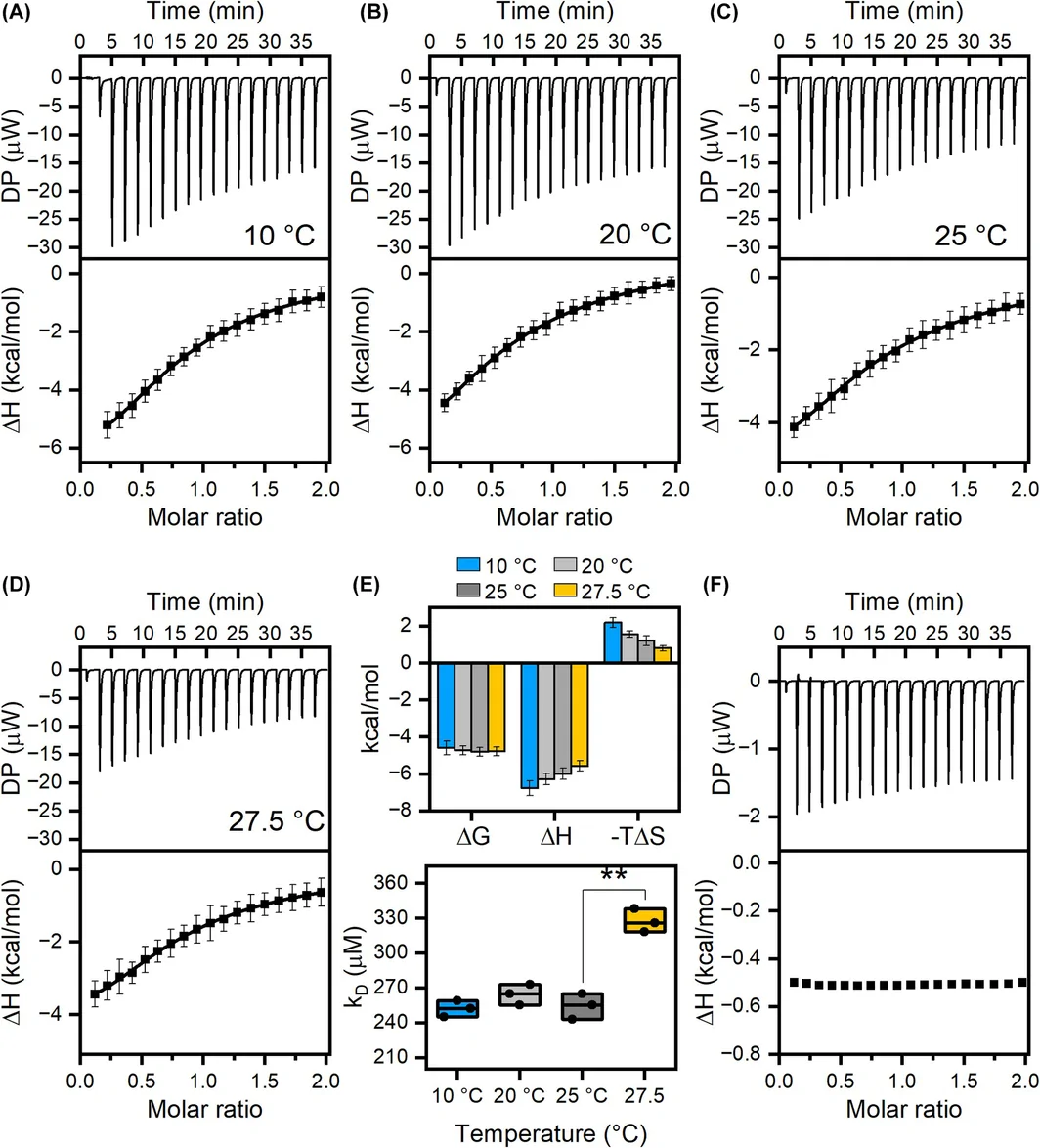
Effects of temperature on the pNPGlc affinity. ITC measurements of M‐GH1^E355A^ were performed at 10 °C (A), 20 °C (B), 25 °C (C), and 27.5 °C (D) in PB with a protein concentration of 500 μm and a pNPGlc concentration of 5 mm in the syringe. Upper panels: binding curves; one representative curve is shown for each titration (*n* = 3). Bottom panels: integrated data corrected for heat of dilution. (E) Thermodynamic parameters and *K*_d_ values determined by ITC measurement performed at different temperatures. Error bars show the standard deviation (*n* = 3), and the horizontal line indicates the mean value. Statistical analyses were performed using an unpaired two‐tailed Student's *t*‐test, **p < 0.01. (F) ITC measurements of Pp‐GH1^E356A^ were performed at 35 °C in PB with a protein concentration of 700 μm and a pNPGlc concentration of 7 mm in the syringe.

ITC measurements also provide valuable insight into the effect of temperature on substrate affinity (Fig. 2B). Between 5 and 25 °C, *K*_M_ kept constant (average value: 340.0 ± 7.2 μm), while it slightly increased at 27.5 °C (407.5 ± 5.3 μm). Notably, at temperatures > 30 °C the *K*_M_ value became 1.5 times higher than that recorded at *T*_*opt*_ (Fig. 2B). This observation prompted further investigation into the binding affinity between M‐GH1 and its substrate in the temperature range 10–30 °C. The dissociation constant (*K*_d_) and binding parameters were determined by titrating the catalytically inactive mutant M‐GH1^E355A^ with pNPGlc. This mutant is essential for studying binding thermodynamics, as it allows the measurement of heat variations solely due to substrate binding, without interference from catalysis. Thermograms obtained at 30 °C were noisy and showed low differential power, suggesting a very low affinity for the substrate. Therefore, our analyses focus on the thermograms obtained in the temperature range 10–27.5 °C. The titrations yielded exothermic binding curves with a 1 : 1 stoichiometry at all tested temperatures (Fig. 3A–D). The binding curves were fitted with a single site binding model, indicating that the apparent *K*_d_ remained stable in the temperature range 10–25 °C (252.2 ± 7.0 μm) and showed a significant increase at 27.5°C (Fig. 3E). Across the temperature range of 10–27.5 °C, the change in binding ΔG was not statistically significant, due to a redistribution of the apparent enthalpy (ΔH) and entropy contribution (TΔS) (Fig. 3E). In summary, the findings from two orthogonal approaches indicate that changes in substrate affinity are indicative of heat‐induced structural changes occurring at temperatures close to *T*_*opt*_.

### Pp‐GH1 exhibits mesophilic properties, and its inactivation is due to heat‐induced denaturation

**Fig. 4:**
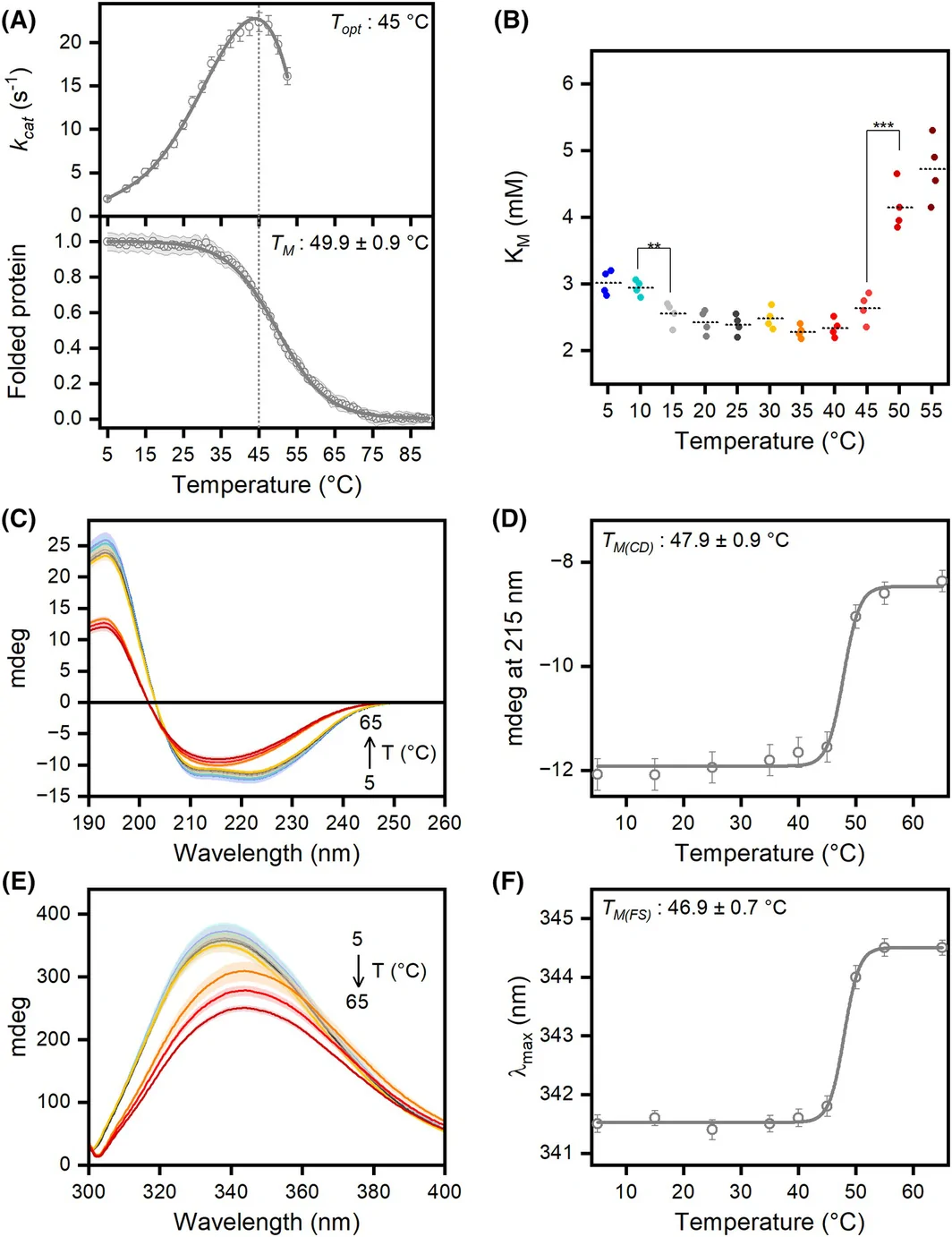
Effects of temperature on the function and structure of Pp‐GH1. Effects of temperature on *k*_*cat*_ (A, upper panel) and *K*_M_ (B) of Pp‐GH1 as determined by ITC analysis. Reactions were performed in a 25 mm phosphate buffer (pH 7.0) using pNPGlc as a substrate (final concentration, 5.0 mm) at an injection rate of 35 μL·s^−1^. The thermal stability of Pp‐GH1 (A, bottom panel) was determined by CD spectroscopy. Ellipticity values were recorded at 215 nm for Pp‐GH1 during heating from 5 to 90 °C. The initial CD signal was considered to be 100% for normalization. Effects of temperature on the structure of M‐GH1 as determined by CD spectroscopy (C, D) and intrinsic fluorescence spectroscopy (E, F). CD spectra (C) and fluorescence emission spectra (E) of Pp‐GH1 measured after ITC analyses were performed by injecting 25 mm phosphate buffer (pH 7) at an injection rate of 35 μL·s^−1^ instead of pNPGlc. The thermal denaturation plot was obtained by plotting the CD signal at 215 nm (D) or the wavelength of the maximum fluorescence emission peaks (F) as a function of temperature. Experiments were performed in quadruplicate, and the error bars or shaded areas indicate the standard deviation (*n* = 4). Statistical analyses were performed using an unpaired two‐tailed Student's *t*‐test, **p < 0.01, ***p < 0.001.

The effects of temperature on the catalytic and structural features of the mesophilic enzyme were investigated using the same experimental setup previously described for M‐GH1. Pp‐GH1 retained 9.0% of its activity at 5 °C (*k*_*cat*_: 2.0 ± 0.3, Fig. 4A), with a *T*_*opt*_ of 45 °C (*k*_*cat*_: 22.1 ± 0.7) and a *T*_*M*_ of 49.9 ± 0.9 °C (Fig. 3A), resulting in a *T*_*G*_ of 4.9 °C. This value is consistent with the average *T*_*G*_ of other mesophilic enzymes, suggesting that enzyme inactivation coincides with the loss of secondary structure [11]. This is further confirmed by the similar *T*_M(CD)_ (47.9 ± 0.8 °C) and *T*_M(FS)_ (46.9 ± 0.7 °C) values obtained from samples analyzed after ITC measurements (Fig. 4C–E).

To evaluate the effects of temperature on substrate affinity, only *K*_M_ values were considered. This was due to the low affinity between a catalytically inactive Pp‐GH1^E356A^ variant and pNPGlc (Fig. 3F), which hindered the ITC titration experiments. The K_M_ values remained constant in the temperature range 5–45 °C (average value: 2.4 ± 0.2 mm) with a slight increase at temperatures < 10 °C (Fig. 4B), suggesting reduced substrate affinity at low temperatures. At 50 °C, K_M_ value increased 1.5‐fold compared to that at *T*_*opt*_ (45 °C), likely due to heat‐induced conformational changes. Both ITC and spectroscopic analyses indicate that M‐GH1 exhibits approximately tenfold greater affinity for pNPGlc compared to Pp‐GH1 at all tested temperatures. This finding is unexpected, given that psychrophilic enzymes typically exhibit greater substrate promiscuity and lower affinity than their mesophilic counterparts due to their increased active site flexibility [1, 2, 3]. The underlying reason for this behavior remains unclear and likely reflects distinct physiological roles of the two enzymes.

### Computational simulations suggest the role of the M‐W‐F triad in temperature adaptation

To gain a deeper understanding of the mechanisms of temperature adaptation of M‐GH1 and Pp‐GH1, we performed MD/EVB simulations of the glycosylation step in both enzymes using cellotriose as substrate. This allows us to use exactly the same EVB parametrization as was earlier determined for the glycosylation step in α‐amylases [27, 28]. The glycosylation step is well‐established as the rate‐limiting step and corresponds to the initial phase of the reaction, leading to the formation of the covalent enzyme‐substrate intermediate [35]. The simulations were carried out in a spherical water droplet of diameter 80 Å that encapsulates the entire protein. For M‐GH1, the calculations predicted an activation free energy at 300 K (27 °C) of 15.2 ± 0.2 kcal·mol^−1^, where the error bars denote the standard error of mean from 30 replicate simulations. This predicted a rate constant of *k*_cat_ = 52·s^−1^ for cellotriose, assuming that the glycosylation step is rate‐limiting also in this case. This agrees well with the measured rate herein for the similar substrate cellobiose of ~42 s^−1^. The simulations of the Pp‐GH1 reaction with cellotriose yielded a higher activation barrier at 300 K by about 1.2 kcal·mol^−1^. The error bars here are, however, twofold higher than for M‐GH1, which is likely due to the significantly larger negative charge of Pp‐GH1 (−24 vs. −13) that slows down the convergence of the free energy calculations.

**Fig. 5:**
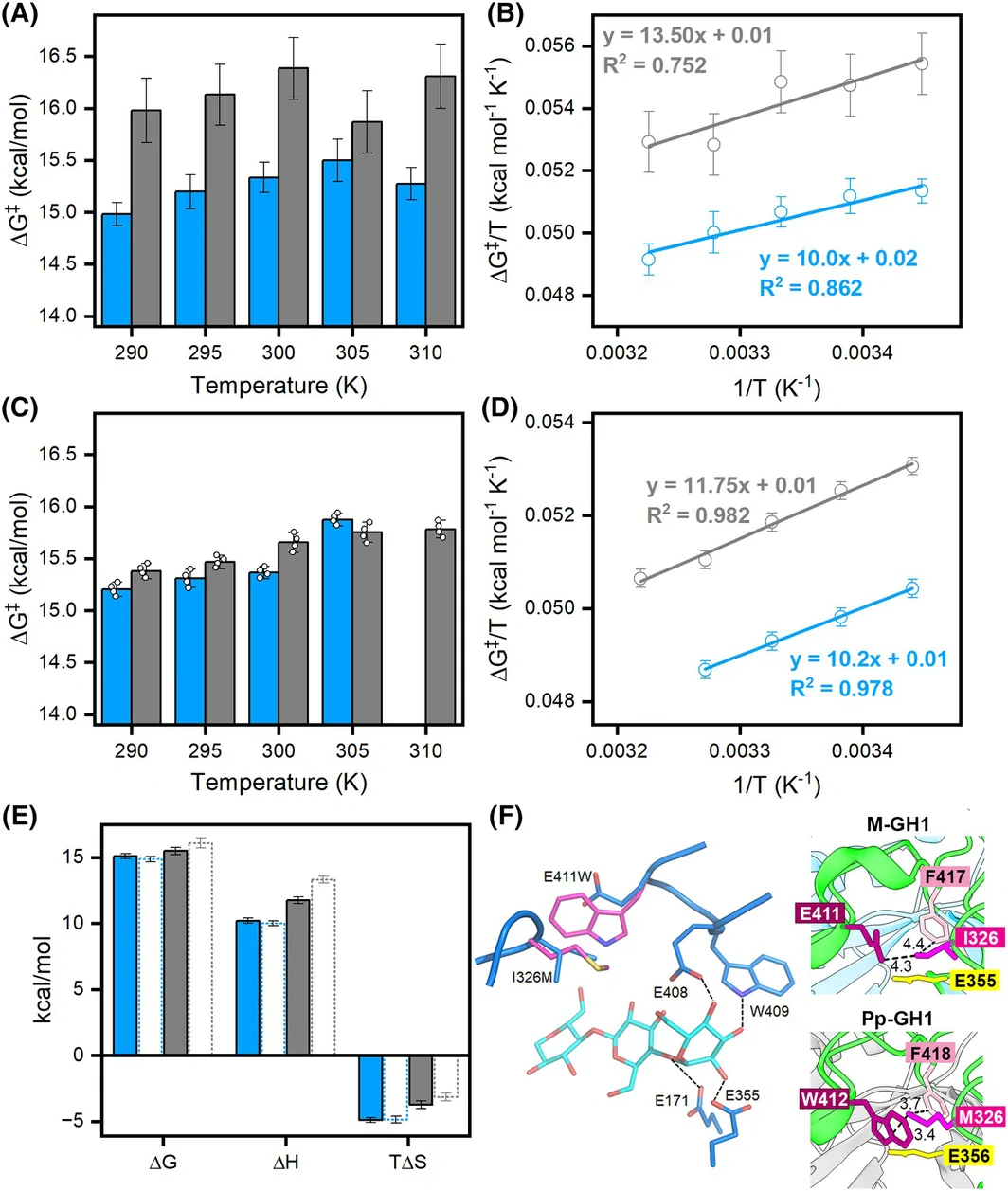
Comparison of experimental and computational thermodynamic parameters for M‐GH1 and Pp‐GH1. Calculated activation free energy barriers as a function of temperature (A) and the corresponding Arrhenius plots (B) for the glycosylation reaction in M‐GH1 (in light blue) and Pp‐GH1 (in gray). Error bars 1 s.e.m. from 30 independent simulations for each enzyme at each temperature. Experimental free energy barriers (C) and the corresponding Arrhenius plots (D) determined by ITC analysis using cellobiose as the substrate. Error bars represent standard deviation (*n* = 4). (E) The thermodynamic parameters of M‐GH1 (light blue) and Pp‐GH1 (gray) were calculated using Eq. 1 at 290 K from experimental (solid line) and computational (dashed line) data. Error bars represent standard deviation. (F) On the left: view of the M‐GH1 active site (blue) with the modeled cellotriose substrate (cyan) used in the MD/EVB simulations. The M326 and W412 side chains found in Pp‐GH1 are also shown (purple) and key hydrogen bonds are indicated. The right panels show a zoom of the I‐E‐F triad of M‐GH1 (PDB: 8PUO) and the M‐W‐F triad of Pp‐GH1 (PDB:2O9R), with distances in Å. In green are indicated the loops surrounding the active site. Images were prepared using UCSF Chimera X [31].

The temperature dependence of the activation free energy was further investigated by MD/EVB simulations at 290, 295, 300, 305, and 310 K. The results from these calculations are shown in terms of the predicted free energy barriers and Arrhenius plots in Fig. 5A,B. It is noteworthy that the experimentally derived free energy barriers of 15–16 kcal·mol^−1^ for the cellobiose substrate are well reproduced by these calculations (Fig. 5C,D), which utilize the earlier EVB parametrization for the uncatalyzed cellobiose reference reaction in water. As can be seen, the simulations essentially predicted that M‐GH1 is faster than Pp‐GH1 over the entire temperature range. Hence, the calculations do not predict any optimum rate for M‐GH1, which probably indicates that slower processes that are not captured by the MD/EVB simulations are responsible for the optimum. The calculated Arrhenius plot for M‐GH1 closely aligns with experimental data, even though the highest temperature point appears to be an outlier in Fig. 5A. In contrast, the calculated Arrhenius plot for Pp‐GH1 simulations shows lower accuracy, as can be seen from the *R* ^2^‐values in Fig. 5B. At 290 K, experimental data indicated a difference in ΔG^‡^ of −0.4 kcal·mol^−1^ between M‐GH1 and Pp‐GH1, while MD/EVB simulations yielded a more pronounced difference of −1.2 kcal·mol^−1^ (Fig. 5E). The thermodynamic activation parameters for M‐GH1 (ΔH^‡^ = 10.0 kcal·mol^−1^ and TΔS^‡^ = −5.1 kcal·mol^−1^) closely matched the experimental values (ΔH^‡^ = 10.2 kcal·mol^−1^ and TΔS^‡^ = −4.9 kcal·mol^−1^). For Pp‐GH1, however, the simulated thermodynamic parameters (ΔH^‡^ = 13.5 kcal·mol^−1^ and TΔS^‡^ = −2.7 kcal·mol^−1^) differed slightly from the experimental values (ΔH^‡^ = 11.8 kcal·mol^−1^ and TΔS^‡^ = −3.7 kcal·mol^−1^), probably due to the lower accuracy of the Pp‐GH1 simulations. Overall, our data demonstrates the typical trend for cold‐adapted enzymes, with M‐GH1 exhibiting a lower activation enthalpy accompanied by a larger entropy penalty compared to its mesophilic counterpart.

**Fig. 6:**
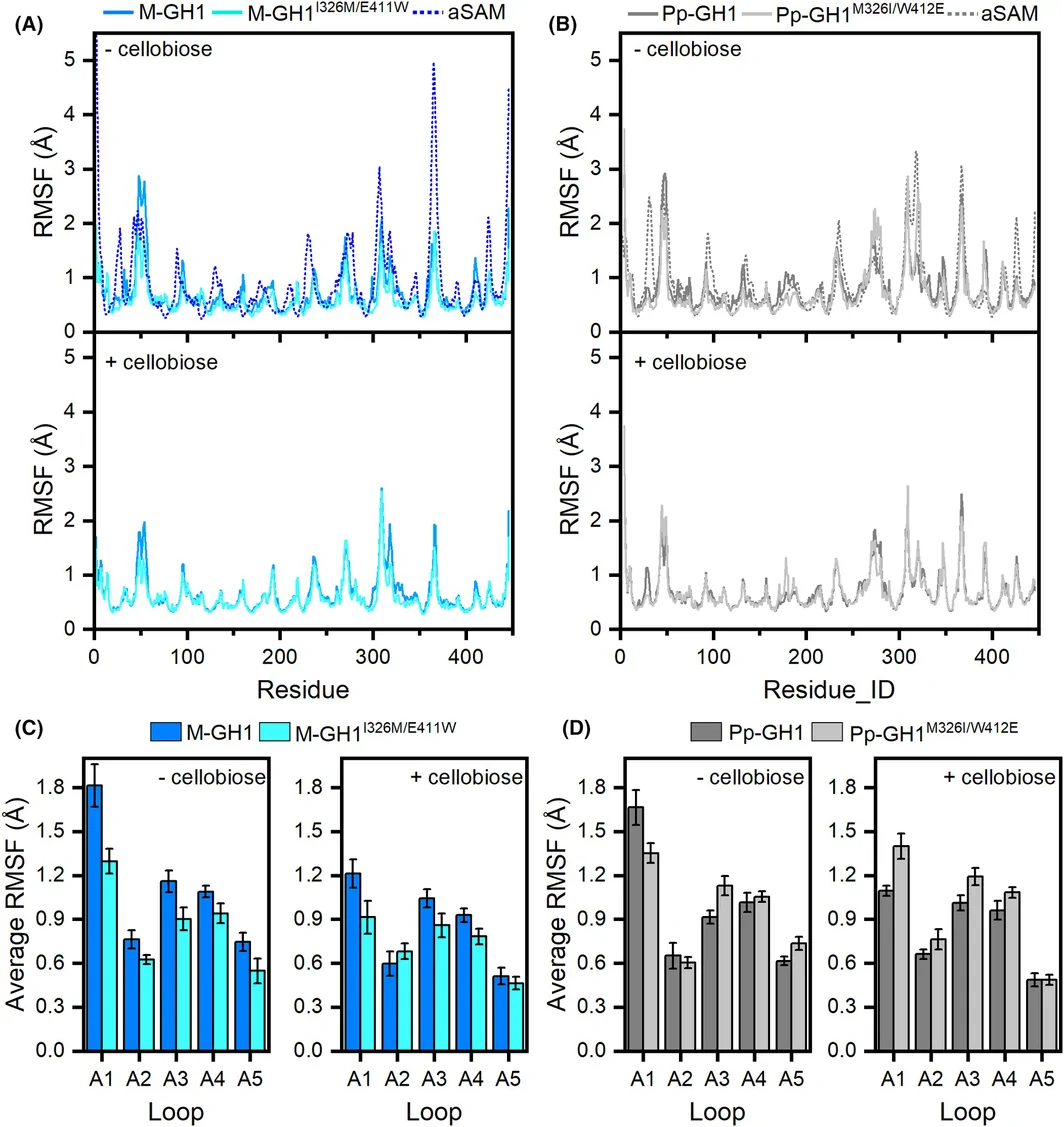
MD simulations of M‐GH1 and Pp‐GH1. RMSF profiles of M‐GH1 (A) and Pp‐GH1 (B) obtained at 300 K in the absence (top panel) and presence of cellobiose (bottom panel). The comparison between aSAMt and classical MD simulations at 300 K for M‐GH1and Pp‐GH1 is shown in the top panel. The Spearman rank correlation coefficient for M‐GH1 was 0.85, whereas that for Pp‐GH1 was 0.79. (C) Flexibility of loops surrounding the active site in M‐GH1 and M‐GH1^I326M/E411W^ variant. (D) Flexibility of loops surrounding the active site in Pp‐GH1 and Pp‐GH1^M326I/W412E^ variant. MD simulations shown are an average of five replicates, error bars represent standard deviation (*n* = 5).

It is not trivial to uncover the structural origin of the differences in the free energy barriers between the two enzymes. It has often shown that cold‐adapted enzymes tend to show a higher degree of conformational flexibility than their mesophilic orthologs [18]. However, in this case, the backbone mobility profiles of M‐GH1 and Pp‐GH1 did not show any major differences at 300 K, both in the absence and presence of cellobiose (Fig. 6A,B). On the other hand, analysis of the average energy differences (ΔU^‡^) between the transition and reactant states revealed some interesting features (Fig. 5B). That is, the electrostatic contribution to the activation enthalpy is clearly more favorable in M‐GH1 than in Pp‐GH1, despite the fact that the active sites are extraordinarily similar. At 300 K (27 °C), this energy term is about 3 kcal·mol^−1^ more favorable in M‐GH1 than in Pp‐GH1 and, notably, its partitioning between substrate interactions with the surrounding protein relative to solvent molecules is rather different. Hence, while the transition state relative to the reactant state in M‐GH1 has more favorable interactions with the protein, in Pp‐GH1 it has more favorable interactions with solvent molecules around the reaction center.

MD/EVB simulations of M‐GH1 revealed that, besides substrate interactions with catalytic residues, E408 and W409 form crucial hydrogen bonds with the terminal sugar moiety (Fig. 5F). These interactions are slightly weaker at higher temperatures, indicating their thermal sensitivity. The loop A5 containing the E408 and W409 residues is highly conserved between the two enzymes, except residue E411 that in Pp‐GH1 is substituted with W412 (Fig. 1C). MD/EVB simulations showed that substrate‐solvent interaction energies are more negative in Pp‐GH1 than in M‐GH1, where they are compensated by substrate–protein interactions. This behavior in Pp‐GH1 can be attributed to interactions among the side chains of W412, M326, and F418, which appear to provide tighter packing and potentially offer more efficient shielding of the bound substrate from the solvent. In M‐GH1, at the analogous positions, only the F417 residue is conserved (corresponding to F418 in Pp‐GH1), while W and M are replaced by E411 and I326, respectively (Fig. 5F).

To investigate the structural roles of these residues, we conducted MD simulations at 27 °C on M‐GH1, Pp‐GH1, and their swapped triad variants, namely M‐GH1^I326M/E411W^ and Pp‐GH1^M326I/W412E^, both in the absence and presence of cellobiose. Grafting the mesophilic M‐W‐F triad into M‐GH1 resulted in a slight reduction in overall flexibility, regardless of the substrate (Fig. 6A). Specifically, the mesophilic triad slightly rigidified all active site loops in the absence of cellobiose, whereas with substrate, rigidity was localized to loops A1, A3, and A4 (Fig. 6C). In contrast, introducing the psychrophilic I‐E‐F triad into Pp‐GH1 slightly increased global flexibility in the absence of substrate (Fig. 6B), mainly affecting the loops A3 and A5. Upon cellobiose binding, the psychrophilic triad enhanced the flexibility of loop A4 and loops A3 and A5, although the global RMSF values remained largely unchanged (Fig. 6B,D).

### Role of the triad M‐W‐F in the stabilization of M‐GH1 active site and in temperature adaptation of GH1 family

To elucidate the role of the M‐W‐F interactions in active site stabilization, we generated three variants of M‐GH1: M‐GH1^I326M^, M‐GH1^E411W^, and the double mutant M‐GH1^I326M/E411W^. Among these, M‐GH1^I326M^ was produced at very low levels not suitable to its functional and structural characterization, while M‐GH1^E411W^ and M‐GH1^I326M/E411W^ were analyzed by ITC for their thermal activity and stability by using the experimental design previously described.

**Fig. 7:**
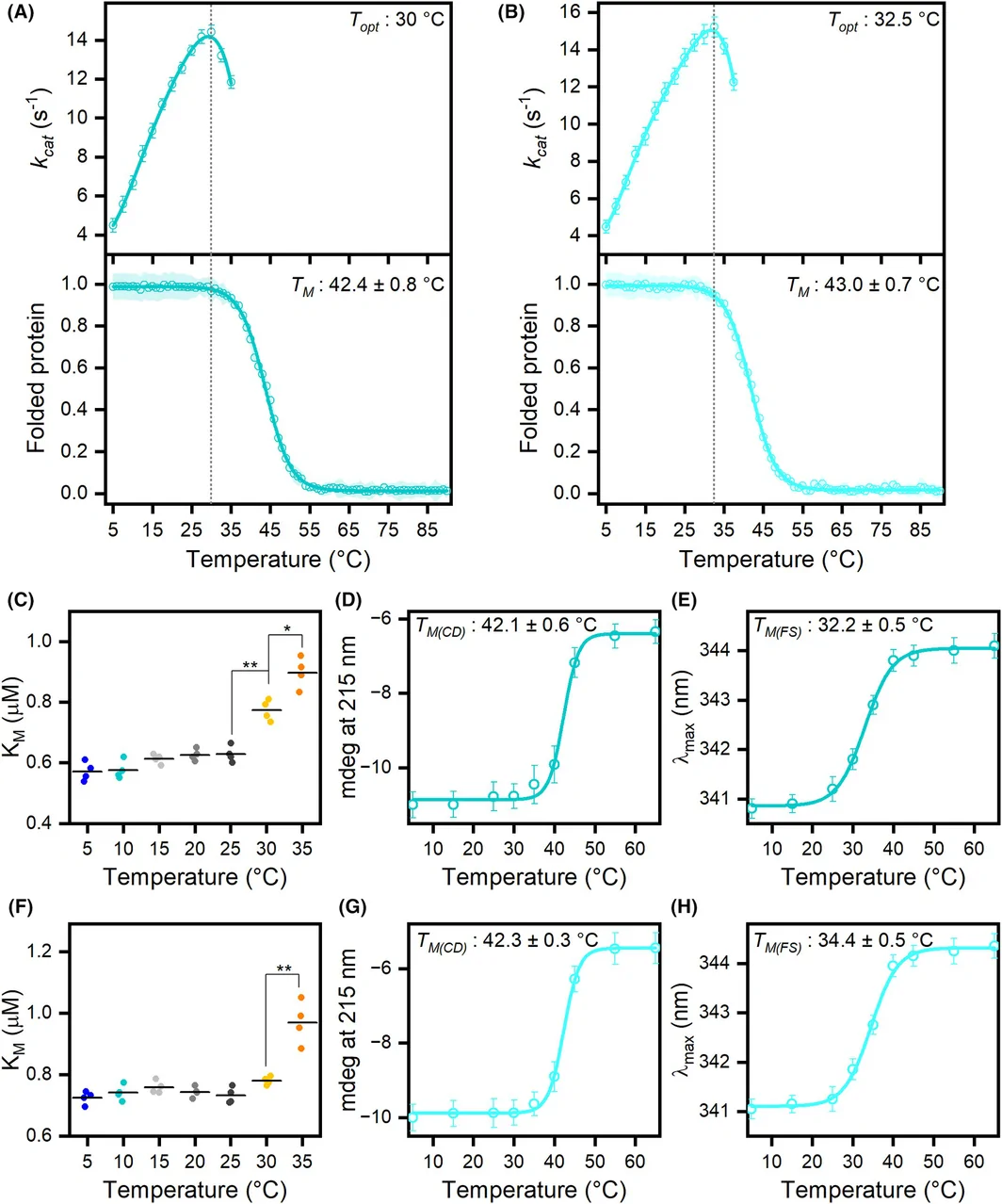
Effects of temperature on the function and structure of M‐GH1^E411W^ and M‐GH1^I326M/E411W^. Effects of temperature on *k*_*cat*_ and K_M_ of M‐GH1^E411W^ (A upper panel and C) and M‐GH1^I326M/E411W^ (B upper panel and F) determined by ITC analysis. The thermal stability of M‐GH1^E411W^ (A, bottom panel) and M‐GH1^I326M/E411W^ (B, bottom panel) were determined using CD spectroscopy. Ellipticity values were recorded at 215 nm during heating from 5 to 90 °C. The initial CD signal was considered 100% for normalization. Thermal denaturation plots of M‐GH1^E411W^ (D and E) and M‐GH1^I326M/E411W^ (G and H) were obtained by plotting the CD signal at 215 nm or the wavelength of the maximum fluorescence emission peak as a function of temperature. CD and intrinsic fluorescence spectra were recorded after ITC measurements as previously described. Experiments were performed in quadruplicate, and the error bars or shaded areas indicate standard deviation (*n* = 4). Statistical analyses were performed using unpaired two‐tailed Student's *t*‐test, *p < 0.05, **p < 0.01.

**Fig. 8:**
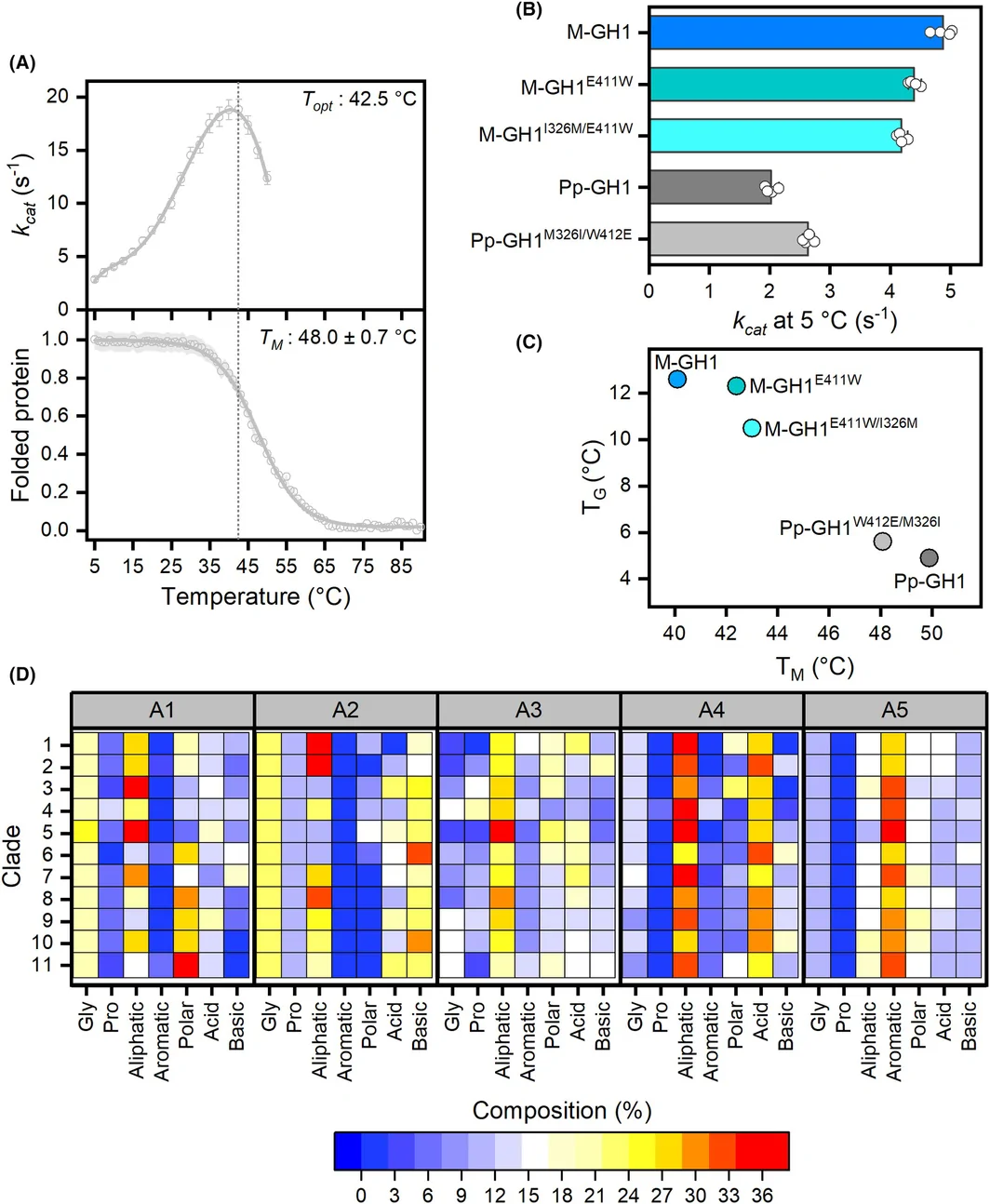
Temperature effects on the function and structure of Pp‐GH1^M326I/W412E^ and loop analysis. (A) Effect of temperature on *k*_*cat*_ (upper panel) and thermal stability (lower panel) of Pp‐GH1^M326I/W412E^. Kinetic parameters were determined by ITC analysis in PB using 5.0 mM pNPGlc (injection rate: 35 μL·s^−1^). Thermal stability was monitored by CD spectroscopy at 215 nm during heating from 5 to 90 °C; the initial signal was normalized to 100%. (B) Specific activity at 5 °C of wild‐type M‐GH1 and Pp‐GH1 compared to their respective variants (M‐GH1^E411W^, M‐GH1^I326M/E411W^, and Pp‐GH1^M326I/W412E^). Error bars represent standard deviation (*n* = 4). (C) Relationship between *T*_M_ and *T*_G_ of M‐GH1, Pp‐GH1 and their variants. (D) Heat maps showing the amino acid composition of loops surrounding the active site for the clades identified in the phylogenetic analysis (see Fig. 9).

In the single mutant, the E411W substitution improved the thermal stability of M‐GH1 without affecting the *T*_*G*_, resulting in a *T*_*opt*_ of 30 °C and a *T*_*M*_ of 42.4 ± 0.8 °C. This likely stems from increased active site rigidity, reducing the substrate affinity (K_M_: 0.6 mM) while preserving the local unfolding events responsible for enzyme inactivation (Fig. 7A,C–E). In contrast, in the double mutant M‐GH1^I326M/E411W^, where the M‐W‐F triad was restored in the M‐GH1 active site, the *T*_*M*_ (43.0 ± 0.7 °C) was comparable to that of the single mutant. Notably, the double mutant exhibited a higher *T*_*opt*_ (32.5 °C) than the M‐GH1^E411W^ single variant. A similar trend was also observed by CD and fluorescence spectroscopy on samples analyzed after ITC measurements (Fig. 7B,D–G). Both M‐GH1 variants remain active at low temperatures, retaining approximately 30% of their activity at 5 °C, which is lower than that of the wild‐type enzyme. It is interesting to note that the double mutant exhibits lower substrate affinity compared to the M‐GH1 and M‐GH1^E411W^ variants. The psychrophilic I‐E‐F triad was grafted into Pp‐GH1 via site‐directed mutagenesis, yielding the Pp‐GH1^M326I/W412E^ variant. In this variant, both *T*_*M*_ (48.0 ± 0.7 °C) and *T*_*opt*_ (42.5 °C, *k*_*cat*_: 18.8 ± 0.5·s^−1^) were slightly lower than those of the wild‐type enzyme (Fig. 8A). At 5 °C, Pp‐GH1^M326I/W412E^ retained 13.8% of its activity (2.6 ± 0.1·s^−1^), slightly higher than the wild‐type but approximately half that of M‐GH1 and its variants (Fig. 8B). To analyze mutation effects, we plotted *T*_*G*_ against *T*_*M*_ for all the enzymes described in this work (Fig. 8C). As expected, the two extremes are represented by M‐GH1 and Pp‐GH1. The E411W substitution increased the *T*_*M*_ while maintaining the *T*_*G*_ constant, suggesting that the steric hindrance of W increases the active site rigidity but the interactions with I326 are suboptimal to affect the *T*_*G*_. Establishing the M‐W‐F triad in the M‐GH1 active site, similar to that in Pp‐GH1, optimized these interactions and decreased the *T*_*G*_ value. In contrast, grafting the psychrophilic residues into Pp‐GH1 slightly increased the flexibility of loops A3 and A5, resulting in a slightly lower *T*_*M*_ and an increase in *T*_*G*_ (5.5 °C for Pp‐GH1^M326I/W412E^ vs. 4.9 °C for Pp‐GH1). The modest magnitude of this reduction suggests that epistatic effects within the mesophilic scaffold buffer the impact of the psychrophilic triad, highlighting the complexity of temperature adaptation [36]. Taken together, our results suggest that the M‐W‐F triad stabilizes loops A3 and A5 and may be crucial for hot adaptation in the GH1 family members.

**Fig. 9:**
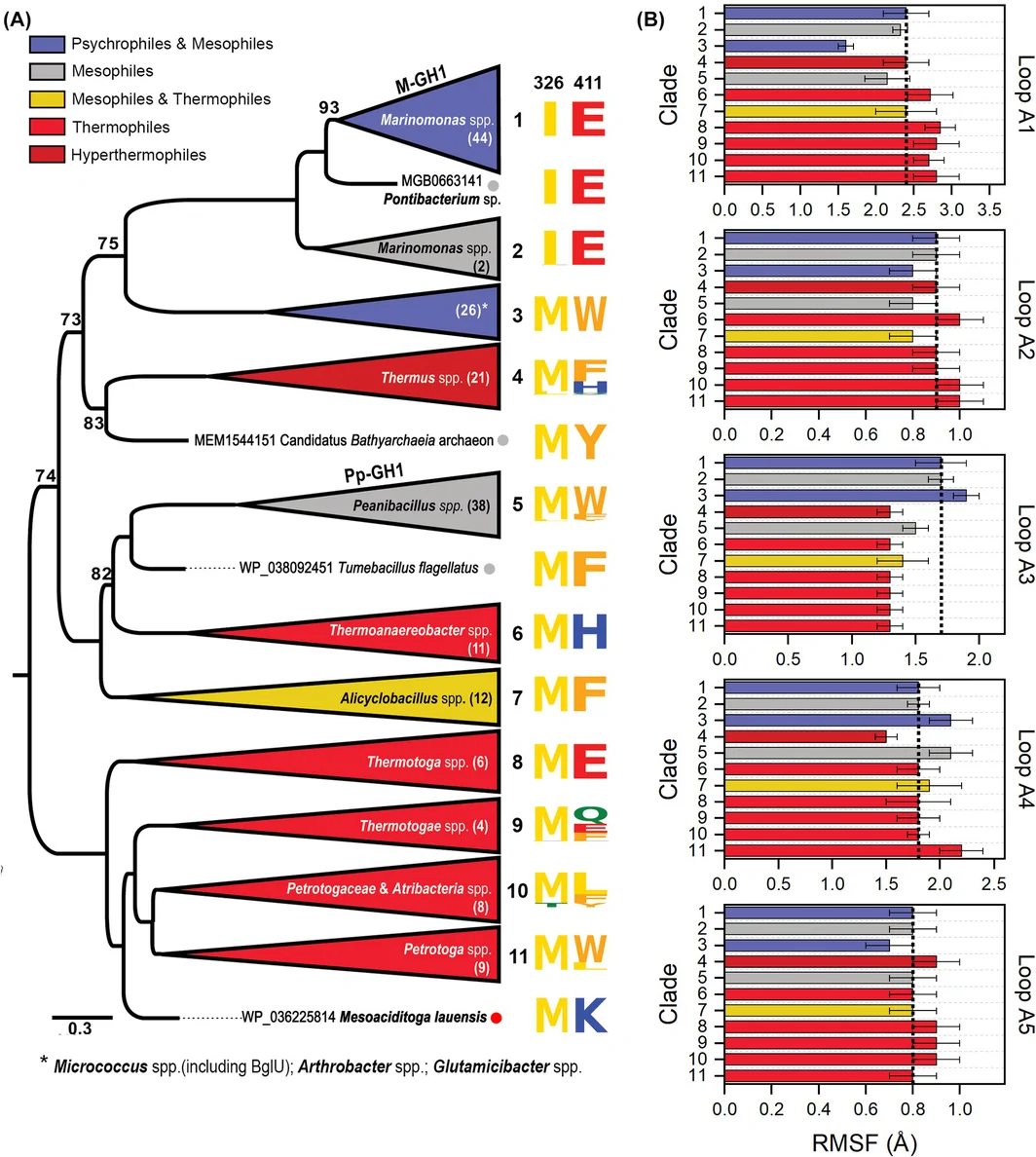
Conservation of residues I326 and E411 in the GH1 family. (A) Phylogenetic analysis of M‐GH1, Pp‐GH1, and evolutionarily related GH1 enzymes from psychrophilic, mesophilic, and thermophilic organisms. To aid visualization, some subtrees were collapsed. Sequence accession numbers are provided in Table S1, and the number of sequences in each clade is indicated in brackets. The number of clades are labeled in bold, and residue conservation at positions 326 and 411 in M‐GH1 is illustrated by sequence logos. Phylogenetic analysis was performed using IQ‐Tree software [38]. (B) Backbone RMSF of GH1 active site loops from aSAMt sampling at 320 K. Values are reported averaged for each clade, error bars represent standard deviation.

To investigate the role of these three residues in the thermal adaptation of the GH1 family, we combined evolutionary analysis with an assessment of the flexibility of loops surrounding the active sites of a dataset of homologs of M‐GH1 and Pp‐GH1. The resulting rooted phylogenetic tree (Fig. 9A) shows three main lineages: the first comprises sequences from psychrophilic (including M‐GH1), mesophilic and hyperthermophilic enzymes (clades 1–4); the second contains sequences from mesophilic (including P‐GH1) and thermophilic enzymes (clades 5–7); and the third consists exclusively of sequences from thermophilic enzymes (clades 8–11). Interestingly, psychrophilic enzymes are distributed across two clades containing psychrophilic and mesophilic organisms rather than being clustered together. Well‐characterized cold‐active BglU from *Micrococcus antarcticus* [37] belongs to clade 3, whereas M‐GH1 belongs to clade 1.

Analysis of the M‐W‐F residue conservation (logo diagram in Fig. 9A) reveals a complex pattern. As the F residue is universally conserved across the GH1 family due to its role in substrate binding, we focused on the positions corresponding to W and M. The M residue is highly conserved, except in clades 1 and 2, which predominantly contain enzymes from *Marinomonas* species. However, only M‐GH1 has been biochemically characterized, limiting speculation on their thermal behavior. The W position exhibits the least conservation; it is conserved in cold‐active enzymes containing clade 3 and replaced by other aromatic residues (Y and F) in several clades. In clades 1, 2, 8, and 9, the W residue is substituted by E. This complex pattern prompted an investigation into the flexibility of the active site surrounding loops using aSAM, a latent diffusion deep learning model trained on MD data to generate heavy atom protein ensembles. Specifically, the aSAMt variant was employed, as it was trained on MD simulations at different temperatures and captures temperature‐dependent ensemble properties [39]. The RMSF values obtained with aSAM at 300 K for M‐GH1 and Pp‐GH1 correlated well with RMSF profiles obtained by classic MD simulations (Fig. 6). The average backbone RMSF of loops A2, A4, and A5 showed no significant variation, whereas the flexibility of loop A3 was strongly correlated with thermal behavior (Fig. 9B). Loop A3 was significantly more flexible (average RMSF ≈ 1.7 Å) in cold‐active enzymes containing clades 1 and 3 than in mesophilic and thermophilic clades (average RMSF ≈ 1.3 Å). Due to the high sequence heterogeneity of these loops, direct comparison across clades was challenging; therefore, we analyzed their amino acid composition and length (Fig. 8D and Table 1). Except for loop A3 in clade 3, which is twice as long as that in the other clades, the loops exhibit similar lengths across all clades (Table 1). Regarding composition, no clear temperature‐related trend emerged, suggesting that thermal adaptation likely involves multiple strategies and requires further investigation. We focused on the mechanisms modulating loop A3 flexibility and identified at least three distinct strategies: (i) In clades 1 (M‐GH1), 5 (Pp‐GH1), and 11, loop A3 has a similar length and composition. However, the M‐W‐F triad remains conserved in clades 5 and 11, potentially playing a crucial role in stabilizing the active site loop. In contrast, its switch to I‐E‐F in clade 1 may be related to increased flexibility of the enzyme. (ii) Clade 3, containing psychrophilic enzymes, achieves high loop A3 flexibility by doubling its length while retaining a ‘mesophilic‐like’ M‐W‐F triad and high proline content, a mechanism previously reported to explain the cold activity of BglU [37]. (iii) Thermophilic clade 8 exhibits rigidity due to a pronounced enrichment of proline residues (12%) compared to psychrophilic clade 1 (1.5%) and mesophilic clade 5 (3.7%). This proline‐mediated rigidity strategy is also observed in other thermophilic clades (4, 7, 9, and 10), where the proline content ranges from 11% to 20%. In these clades the M in the triad is conserved while the W is not. Overall, these findings suggest that thermal adaptation in GH1 enzymes is primarily governed by the modulation of loop A3 flexibility and the properties of the surrounding interacting residues, especially those within the triad.

**Table 1:** Length of loops surrounding the active sites. Values are average for sequences belonging to the same clade. Standard deviation is not reported when it is zero.

	Loop				
	A1	A2	A3	A4	A5
1	14	9	25.9 ± 0.3	15	18
2	14	9	27	16	18
3	14	9	46	15	18
4	14	9	24.9 ± 0.4	16 ± 0.2	18
5	14	9	27	15	18
6	14	9	26	16	18
7	14	9	27.1 ± 0.3	15	18
8	14	9	25.5 ± 0.6	15.7 ± 0.5	18
9	14	9	25	15	18
10	14	9	25	15	18
11	14	9	25	15	18

In conclusion, the aim of this study is to investigate the molecular mechanisms of temperature adaptation in M‐GH1, a cold‐active enzyme isolated from the Antarctic bacterium *Marinomonas* sp. ef1 by comparing its biochemical features with those of Pp‐GH1, a mesophilic homolog. These enzymes share 45.4% sequence identity and exhibit high conservation of the active site and overall structure. Our findings suggest that the thermal inactivation of M‐GH1 is due to local unfolding events since it coincides with the loss of tertiary structure, which precedes the loss of enzyme secondary structure. In contrast, the mesophilic Pp‐GH1 encounters simultaneously thermal inactivation and loss of both secondary and tertiary structures. MD/EVB simulations further suggest that M‐GH1 inactivation is further influenced by weaker substrate interactions. This mechanism, which could be called ‘local melting’, primarily involves hydrogen bonds and has been observed in other cold‐active enzymes [27, 28, 40]. Overall, these data support previously reported general mechanisms explaining the anomalous *T*_*opt*_ profile of psychrophilic enzymes compared to their mesophilic and thermophilic counterparts [12, 13]. Additionally, M‐GH1 exhibits a lower activation enthalpy accompanied by a larger entropy penalty compared to its mesophilic counterpart, a characteristic trait of cold‐active enzymes [4, 12, 13].

The main structural difference between M‐GH1 and its mesophilic counterpart appears to be a triad comprising M326‐W412‐F418, which stabilizes loops A3 and A5 in Pp‐GH1. Grafting this M‐W‐F triad in M‐GH1 through rational mutagenesis yielded a more stable variant, as evidenced by increased *T*_*M*_ and *T*_*opt*_ values. In contrast, reciprocal mutations in Pp‐GH1 slightly decrease both *T*_*M*_ and *T*_*opt*_ values. Evolutionary analysis indicates that the M‐W‐F triad is not conserved within the GH1 family and may be one of the main strategies that evolution has selected to modulate the flexibility of the loops surrounding the active site.

In conclusion, our work supports the current view that the temperature adaptation of enzymes is not governed by a single feature, but rather by an interplay of several structural elements, including surface loops that do not necessarily belong to the catalytic center [22, 23, 24, 25, 26, 27, 28, 40].

## Methods

### Cloning and mutagenesis

The expression vector pET‐21[M‐GH1] has been described previously [29]. The gene encoding Pp‐GH1 (UNIPROT: P22505) was sequence‐optimized for expression in *Escherichia coli* cells, chemically synthesized (GenScript, Piscataway, NJ, USA), and cloned in‐frame with a C‐terminal 6xHis‐Tag into the pET‐21a vector (EMD, Millipore, Billerica, MA, USA) between *Nde*I and *Xho*I sites to obtain pET‐21 [Pp‐GH1]. Mutagenesis was performed by QuickChange® PCR using the primers listed in Table 2. Reactions were performed using Q5® High‐Fidelity DNA Polymerase (New England BioLabs, Ipswich, MA, USA) and Eppendorf Master‐cycler (Eppendorf, Hamburg, Germany) under the following conditions: one cycle (98 °C for 2 min), 25 cycles (98 °C for 10 s, annealing temperature – *T*_A_ – for 25 s and 72 °C for 3 min) and a final cycle at 72 °C for 3 min. Mutations were verified by bidirectional DNA sequencing.

**Table 2:** List of primers used in this work.

		Mutation	*T*_A_ (° C)
FW	GCGTGATGAGCTGGTTAATGGCCAAAT	Pp‐GH1^E356A^	68
RV	ATAGCCGCGCCGTTCGCGGTGATCAAAATCGG	Pp‐GH1^E356A^	
FW	GAGCCGGTTACCGATATTGGCTGG	Pp‐GH1^M326I^	64
RV	TTCCATGTGAACTTGTTCAACCTGGTC	Pp‐GH1^M326I^	
FW	AATGGGCAGAAGGCTACAGCAAG	Pp‐GH1^W412E^	62
RV	CAAAGTTATCCAGAAAGCTCCAAAC	Pp‐GH1^W412E^	
FW	GCGCGGACCAGATCATTGATGGCG	M‐GH1^E355A^	70
RV	ACGCCGCACCGTTTGCGGTAATATAGATCGGC	M‐GH1^E355A^	
FW	TTGAGTGGGCGTGGGGCTATAGC	M‐GH1^E411W^	68
RV	AGTTATCCATCAGGCTCCACGCG	M‐GH1^E411W^	
FW	CACCGACATGGGCTGGGAAATTGCG	M‐GH1^I326M^	69
RV	TACTCAACGGTCTGGCTGTTTTTCACG	M‐GH1^I326M^	

### Recombinant protein production and activity assays

Recombinant enzymes were produced in *Escherichia coli* BL21 (DE3) cells, extracted, and purified as previously described [29]. The fractions with the highest enzyme concentrations were subsequently subjected to buffer exchange by gel filtration on PD10 columns (GE Healthcare, Little Chalfont, UK) in a final phosphate buffer (PB, phosphate buffer 25 mm, pH 7.0). Bradford protein assay was used to determine protein concentrations. Enzyme activity was assessed at 25 °C using para‐nitrophenyl β‐D‐glucopyranoside (pNPGlc) as the substrate. After 3 min, the reaction was stopped by adding an equal volume of 1 M sodium carbonate buffer, pH 11.0, and the absorbance was measured at a wavelength of 405 nm (molar extinction coefficient: 18.6 mm ^−1^·cm^−1^) using a Jasco V‐770 UV/NIR spectrophotometer (JASCO Europe, Lecco, Italy).

### Isothermal titration calorimetry (ITC)

ITC experiments were performed using a MicroCal PEAQ‐ITC microcalorimeter (Malvern Panalytical). To ensure a perfect solvent match, pNPGlc was dissolved in 25 mm PB (pH 7), which is the same buffer used for the enzyme solution.

In the activity assays, recombinant enzymes (500 nm) were added to the sample cell and pNPGlc (40 mm) was loaded in the injection syringe. Measurements were conducted in the single‐injection mode using the following parameters: stirring speed: 750 rpm, initial delay: 60 s, injection volume: 35 μL, injection rate: 35 μL·s^−1^, reference power: 10 μcal·s^−1^; temperature range: 5–40 °C for M‐GH1 and M‐GH1^E411W^, and 5–60 °C for Pp‐GH1. Kinetic parameters (*K*_M_ and *k*_*cat*_) were determined by fitting the experimental data with MicroCal PEAQ‐ITC analysis software (Malvern).

To compare the experimental data with computational simulations, ITC assays were performed as previously described. Cellobiose (50 mM) was used as the substrate at temperatures of 17 °C (290 K), 22 °C (295 K), 27 °C (300 K), 30 °C (305 K), and 37 °C (310 K). The value of ΔG^‡^ at each temperature was calculated using the Arrhenius–Eyring equation (eq. 1):

$$k_{\mathrm{cat}}=\frac{k_{B}\cdot T}{h}\cdot e^{-\frac{{\mathrm{\Delta G}}^{‡}}{\mathrm{RT}}}$$

where *k*_*B*_ is the Boltzmann constant (1.38 × 10^−23^ J·K^−1^), h the Planck constant (6.63 × 10^−34^ J·s), and ΔG^‡^ the free energy of activation.

In the binding assays, M‐GH1^E355A^ or Pp‐GH1^E356A^ (550 μm) was loaded to the sample cell, and pNPGlc (40 mm) was the titrant in the injection syringe. Measurements were conducted in the multiple‐injection mode using the following parameters: stirring speed: 750 rpm; initial delay: 60 s; injection spacing: 110 s; injection duration: 2 s; reference power: 10 μcal·s^−1^; injections: 20 (a single injection of 0.4 μL, followed by 19 injections of 1 μL); temperature: 10, 20, 25, and 27.5 °C for M‐GH1^E355A^, and 35 °C for Pp‐GH1^E356A^. At each temperature, a control titration of pNPGlc against PB buffer was performed, and the resulting thermogram was subtracted from the one collected for enzyme/pNPGlc titration. Binding parameters were determined by fitting the experimental data with MicroCal PEAQ‐ITC analysis software (Malvern). Experiments were performed in quadruplicate.

### Circular dichroism (CD) spectroscopy

CD spectra were recorded on a J815 spectropolarimeter (JASCO Europe) using a 0.1‐cm path length cuvette. Measurements were performed in triplicate with an enzyme concentration of 3–5 μm, 0.1‐nm data pitch, and 10 nm·min^−1^ scanning speed. All spectra were corrected for buffer contribution, averaged from three independent acquisitions, and smoothed using third‐order least‐squares polynomial fitting.

Thermal unfolding was monitored by recording the CD signal at a fixed wavelength of 215 nm in the temperature range of 5–90 °C with a temperature slope of 1 °C per min.

### Effects of ITC measurements on enzyme structure

To study the impact of ITC measurements on enzyme structure, ITC experiments were performed by titrating the enzyme (5 μm) with 25 mm PB (pH 7). The measurements were carried out in single‐injection mode using the parameters previously described to determine enzyme activity. At the end of the ITC measurements, a sample of 200 μL was withdrawn from the sample cell and subjected to CD and intrinsic fluorescence spectroscopy. CD spectra were recorded as previously described. Fluorescence emission spectra were recorded after excitation at 290 nm with a Cary Eclipse Spectrofluorometer (Varian Australia Pty Ltd, Mulgrave, Vic., Australia) using quartz black cuvettes with a 1 cm path length. The slit width was 5 nm for both excitation and emission. Spectra were acquired at 1 nm intervals at a rate of 600 nm·min^−1^ and an averaging time of 0.1 min. Spectra were measured in the 300–400 nm range, corrected for buffer contribution, averaged from three independent acquisitions, and smoothed using a third‐order least‐squares polynomial fit.

The unfolding profile was obtained by plotting either the CD signal at 215 nm or *λ*_max_ of the fluorescence spectra as a function of temperature. The curve was fitted to OriginLab software (OriginLab Corporation, Northampton, MA, USA) using the Boltzmann sigmoidal equation to determine the unfolding melting transition point *T*_M(CD)_ and *T*_M(FS)_.

### Computational methods

#### System preparation

The computational models for M‐GH1 and Pp‐GH1 were based on the crystal structures PDB: 8PUO [29] and PDB: 2O9P [30], respectively. The cellotriose substrate was modeled from that in the inactive mutant structure PDB: 3SCV. Hydrogen atoms were generated, and the systems were solvated in spherical droplets of diameter 80 Å with the Q program [41]. The TIP3P water model [42] was used, and solvent molecules at the sphere boundary were subjected to radial and polarization restraints following the SCAAS model [41, 43]. The OPLS‐AA/M force field [44] was used in all calculations, and reactive EVB potential energy surface parametrization was taken without change from [28].

#### MD/EVB simulations

All MD/EVB simulations were performed with the Q program (v. 5) [41]. Lennard–Jones interactions were truncated beyond 10 Å and long‐range electrostatic interactions beyond this cutoff were treated with the local reaction field multipole expansion method [45], except for interactions involving the substrate and reacting side chains of the protein (cellotriose and the side chains of Glu171 and Glu355), to which no cutoffs were applied. The models were first equilibrated using a stepwise heating scheme to reach the different target temperatures (during 0.52 ns). The systems were then equilibrated for an additional 1.5 ns at the target temperature with reassignment of random velocities from a Maxwellian distribution. The last 1.75 ns of equilibration was run with a 50/50 mixture of the reactant and product state potentials to yield the starting point for free energy perturbation (FEP) calculations of reaction free energy profiles.

After equilibration, MD/EVB free energy perturbation FEP calculations [46, 47] were run starting from the 50/50 mixture and propagated towards reactants and products. Each of these FEP calculations involved 21 discrete windows with 50 ps of sampling in each, yielding 1.05 ns of sampling for each free energy profile. All calculations were repeated 30 times at each temperature with different initial velocities (see above). The temperature dependence of the activation free energy barriers was examined by carrying out the above protocol at five different temperatures (290, 295, 300, 305, and 310 K). The earlier calibrated EVB parameters were used [28], namely the gas‐phase shift and off‐diagonal parameters (∆*α* = 20.6, H_12_ = 153.36 kcal·mol^−1^), which reproduce the energetics of a small reference system in water (cellobiose + two carboxylic side chains) determined by M06‐2X/6–311 + G(2d,2p) DFT calculations with the SMD solvation model.

#### RMSF calculations

MD simulations were carried out on M‐GH1, M‐GH1^I326M/E411W^, Pp‐GH1, and Pp‐GH1^M326I/W412E^. I326M and E411W mutations for the M‐GH1 enzyme and M326I and W412E for Pp‐GH1 were carried out in USCF Chimera software [31, 48] using the Dunbrack rotamer library [49]. Atomic positional fluctuations for the enzymes were assessed by MD simulations with the GROMACS software (v. 2022.4) [47] using the OPLS‐AA/M force field. These calculations were carried out with a dodecahedral box of volume 445.89 nm^3^ solvated by 12 272 SPCE water molecules [50], where the net charge of the two enzymes was neutralized by Na^+^ counterions. The E411W mutation in M‐GH1^I326M/E411W^ decreased the total charge by one; consequently, one counterion was replaced with a water molecule. The opposite adjustments were applied to the Pp‐GH1^M326I/W412E^ system. The equilibration phase was performed in several steps, starting with the steepest descent algorithm for energy minimization. MD equilibration proceeded in ten consecutive steps, starting at 5 K and position restraints of 200 kcal·mol^−1^·A^−2^ on all protein heavy atoms and counterions. During these steps, each lasting 100 ps with a 1 fs integration time, the temperature was gradually increased to 300 K, and the position restraints were reduced to zero while equilibrating the temperature and pressure. Subsequently, equilibration continued for an additional 100 ns, with the first 10 ns performed at a 1 fs time step and the remaining 90 ns at a 2 fs time step. The production run consisted of 400 ns of molecular dynamics with a 2 fs time step, and the trajectories were saved every 200 fs. These simulations were conducted in five replicates, each initiated with randomized initial velocities, continuing the equilibration stage. From these calculations, the RMSF profiles for the Cα atoms were obtained after least‐squares fitting of the α‐helices and β‐sheets of the respective enzyme structure.

### Evolutionary and structural dynamics analysis

Homologous sequences of M‐GH1 and Pp‐GH1 with available 3D structures were retrieved using the FoldSeek server (https://search.foldseek.com/search). The 3D structures of both enzymes (PDB: 8PUO and 2O9P) were used as input against the PDB100 20240101 database, employing the default 3Di encoding. M‐GH1, Pp‐GH1, and the four most similar structural homologs (> 40% identity) were selected to represent the sequence/structure space between. GH1 enzymes with different oligomerization states (e.g., GH1 beta‐glucosidase from *Exiguobacterium antarcticum* B7 [51]) were excluded to avoid introducing additional variables. The selection process also considered sequences from host organisms with varying temperature adaptations.

The sequences of the six PDB structures (8PUO, 2O9P, 5IDI, 6ZIV, 1UG6, 3 W53) were used as seeds for a sequence‐based search against the UniProt Release 2025_03 (https://www.uniprot.org/blast). Other sequences from organisms of the same genus or with at least 65% pairwise global identity were selected and clustered at 98% identity using CD‐HIT version 4.8.1. The resulting 186 centroid sequences were used to study structural dynamics transitions during the evolution of orthologous GH1s from groups of species with different temperature adaptation profiles. This analysis involved first estimating structural dynamics and then determining phylogenetic relationships among these sequences.

Structural dynamics were estimated by calculating RMSF profiles for Cα atoms using the aSAM temperature‐conditioned model (aSAMt, [39]) at 320 K. Default parameters (500 samples per model) were applied, and each structure in the ensemble was least‐squares fitted to the α‐helices and β‐sheets of the input 3D structures. These input structures were obtained from PDB or AFDB (https://alphafold.ebi.ac.uk/) when available; otherwise, they were predicted as monomers using Colabfold 1.5.5 [52] with the deepfold_v1 model [53].

Phylogenetic analyses were conducted using IQ‐Tree v.2.2.2.7 [38] to construct a Maximum Likelihood phylogenetic tree. The GH1 structure from *Trichoderma harzianum* T7 (PDB: 5BWF) was designated as the outgroup. A structure‐based sequence alignment was performed using Foldseek release 8 [54] with the 3D models generated previously.

The model finder routine [55] identified LG + R6 as the optimal evolutionary model, combining the LG model [56] with a FreeRate model [57] of intersite variation using six categories. This selection was based on both Bayesian and Akaike information criteria [58]. Branch support was assessed using 1000 ultrafast bootstrap replicates [59] and transfer bootstrap expectation [60].

## Author contributions

M.M. and J.Å. conceived of and supervised the project and wrote and revised the original manuscript. S.D. carried out the mutagenesis and recombinant production, conducted the experiments, and revised the original manuscript. G.O. performed the MD and MD/EVB simulations and revised the original manuscript. M.O. performed the evolutionary and structural dynamics analyses and revised the original manuscript. M.L. supervised the project and critically revised the manuscript.

## Conflict of interest

The authors declare no conflict of interest.

## Supporting information


**Table S1.** List of the sequences used in phylogenetic analysis.

## Data Availability

The data that support the findings of this study are available from the corresponding author (marco.mangiagalli@unimib.it) upon reasonable request.
