# Supplementary material for: Discovery of a cryptic aminoacidic triad involved in the temperature adaptation of GH1 enzymes
Source: FEBS J. 2026 Mar 29;293(17):5282–302. doi: 10.1111/febs.70515 (PMC13534868; doi:10.1111/febs.70515)
Supplement: Supplementary file 1 [file FEBS-293-5282-s001.pdf]

Supplementary materials for

# Discovery of a cryptic aminoacidic triad involved in the temperature adaptation of GH1 enzymes

Stefania Digiovanni[1], Gabriel Oanca[2], Marco Orlando[1,3], Marina Lotti[1], Johan Åqvist[2]*
Marco Mangiagalli[1]*

[1]Department of Biotechnology and Biosciences, University of Milano-Bicocca, Piazza della Scienza 2, 20126 Milano, Italy

[2] Department of Cell and Molecular Biology, Uppsala University, Biomedical Center, SE-751 24 Uppsala, Sweden

[3] Present address: Laboratory for Environmental and Life Sciences, University of Nova Gorica, Vipavska cesta 13, Nova Gorica 5000, Slovenia

* Corresponding Authors:

**This PDF file includes:**
- Table S1

**Table S1. List of the sequences used in phylogenetic analysis**

| Accession number | | | |
|---|---|---|---|
| 1OD0 | WP_014062104 | WP_113875607 | WP_272642083 |
| 2JIE | WP_014062252 | WP_113918268 | WP_272643752 |
| 3W53 | WP_014277617 | WP_114412717 | WP_275565199 |
| 4BCE | WP_014464757 | WP_115469393 | WP_305166596 |
| 5IDI | WP_014630558 | WP_121567451 | WP_320964467 |
| 6ZIV | WP_015065271 | WP_121957502 | WP_322158290 |
| 8PUO | WP_018885079 | WP_121959833 | WP_324091228 |
| AAF36392 | WP_019907310 | WP_126165003 | WP_337845222 |
| AAM23648 | WP_022875571 | WP_126201129 | WP_338543926 |
| AAZ81839 | WP_024023547 | WP_128104291 | WP_342415218 |
| ADD25173 | WP_025678475 | WP_133503843 | WP_342426942 |
| BAA86923 | WP_025717718 | WP_133560230 | WP_342444653 |
| CAA91220 | WP_028591107 | WP_134159291 | WP_345472733 |
| GAA6755775 | WP_028991588 | WP_134781046 | WP_353513196 |
| GAB2308818 | WP_029390207 | WP_143591954 | WP_353666861 |
| GAB3479068 | WP_036225814 | WP_148834730 | WP_370534021 |
| HAY44393 | WP_038092451 | WP_149596331 | WP_377365831 |
| HOB16872 | WP_042834089 | WP_149619435 | WP_392385580 |
| HOQ51821 | WP_044645649 | WP_151255964 | WP_394183649 |
| HQE24686 | WP_046019638 | WP_161448342 | WP_400384540 |
| HXL02015 | WP_047117969 | WP_161848827 | WP_404289158 |
| MBP9014879 | WP_054971196 | WP_165179221 | WP_404519644 |
| MCD6546068 | WP_058094603 | WP_167440923 | WP_409376331 |
| MCL4408305 | WP_058712256 | WP_168826582 | WP_414677599 |
| MCL6454266 | WP_060700580 | WP_188687316 | WP_417529080 |
| MDD3600937 | WP_062307411 | WP_191595993 | WP_417561439 |
| MDI3472075 | WP_063212205 | WP_195868140 | WP_418138439 |
| MDK2895978 | WP_063334264 | WP_199462154 | WP_421846883 |
| MDK2907252 | WP_068500100 | WP_199469910 | WP_424609700 |
| MDP5057545 | WP_069011258 | WP_201351639 | WP_428006214 |
| MDR6726863 | WP_071214824 | WP_205116415 | WP_428013854 |
| MEM1544151 | WP_071678184 | WP_206696889 | WP_428720587 |
| MEO9654390 | WP_072841863 | WP_206832407 | WP_430048315 |
| MEP2617726 | WP_072966777 | WP_210002161 | WP_430187962 |
| MEP3350081 | WP_075154652 | WP_212916922 | WP_431087276 |
| MET0871403 | WP_076345037 | WP_218817840 | WP_431493610 |
| MFQ3187063 | WP_088489977 | WP_220025867 | XUQ81432 |
| MGB0663141 | WP_093007242 | WP_226000651 | WP_272642083 |
| NVK75665 | WP_094154088 | WP_228189740 | WP_272643752 |
| OWA35684 | WP_096285392 | WP_229523248 | WP_275565199 |
| TPE50033 | WP_101510358 | WP_234507950 | WP_305166596 |
| A0A2T4AR08 | WP_101512845 | WP_244345446 | WP_320964467 |
| WP_006446764 | WP_103064631 | WP_244454949 | |
| WP_008631473 | WP_103066057 | WP_251384551 | |
| WP_009834590 | WP_103078373 | WP_258297178 | |
